# Effect of gastric lavage with hemostasis powder^® ^on upper gastrointestinal bleeding (Conversion of emergency endoscopy to elective endoscopy)

**DOI:** 10.22088/cjim.11.3.304

**Published:** 2020-05

**Authors:** Seyed Musaal-Reza Hosseini, Malihe Dadgar Moghaddam, Samaneh Yazdan Panah, Jamshid Vafaeimanesh

**Affiliations:** 1 Department of Internal Medicine, Faculty of Medicine, Mashhad University of Medical Sciences, Mashhad, Iran; 2 Department of Community Medicine, Faculty of Medicine, Mashhad University of Medical Sciences, Mashhad, Iran; 3 Student Research Center, Mashhad University of Medical Sciences, Mashhad, Iran; 4 Clinical Research Development Center, Qom University of Medical Sciences, Qom, Iran; 5 Gastroenterology and Hepatology Disease Research Center, Qom University of Medical Sciences, Qom, Iran

## Abstract

**Background:**

From many years ago, gastric lavage has been one of the main pillars of the classic treatment for upper gastrointestinal bleeding (UGIB). The present study investigated the effect of gastric lavage with hemostatic powder on the UGIB complications.

**Methods:**

This clinical trial study was performed on 54 patients who referred to the emergency department during 2017-2018. The subjects were divided into two groups (n=27 per group). Gastric lavage with saline and hemostasis powder^® ^was performed in the control and experimental groups, respectively. The patients' information was collected and analyzed using SPSS software Version. 18. The significance level was set to p<0.05%.

**Results:**

In this study,59.2% and 18.5% of the patients in the gastric lavage with Hemostasis Powder® and saline required no treatment during the early endoscopy, respectively (*p*=0.002). The duration of endoscopy was shorter in the experimental group (p=0.001), (4.83±8.04 hours vs.6.73±14.12 hours, respectively) (*p*=0.001). Moreover, the gastric lavage with Hemostasis Powder^® ^significantly improved the quality of endoscopy .There was no difference between the two groups in terms of their need for blood transfusion (*p*=0.4).

**Conclusion:**

Gastric lavage with hemostasis powder^® ^is a useful measure in the primary treatment of patients with UGIB and can convert emergency therapeutic endoscopy to diagnostic elective endoscopy with higher quality.

An initial evaluation of a patient with UGIB includes the investigation of the patient’s history, physical examination, paraclinical tests, and, in some cases, gastric lavage using a nasogastric tube. Nasogastric intubation, however, is associated with considerable pain and suffering in patients as such that some authors call it modern torture. On the other hand, it has been associated with complications, including stomach rupture (1-2), in some cases. Moreover, some studies have not proven the usefulness of nasogastric intubation (3). For example, 632 patients with gastrointestinal bleeding were examined in a retrospective study. There was no difference among the patients with nasogastric (NG) lavage, and patients with similar characteristics that did not experience NG lavage in outcomes. However, NG lavage was associated with shorter time to endoscopy. In this regard, there was no difference between those who underwent the NG lavage and the control group in terms of mortality rate, length of stay, duration of surgery, or need for blood transfusions. Similarly, the results of randomized trial on 280 patients with UGIB showed no difference between patients with the NG lavage and the control group in terms of re-bleeding or mortality rate (4).

One of the main reasons for the use of the NG lavage is improved endoscopic vision; however, this advantage is now questioned based on the intravenous injection of erythromycin. For example, the results of a study showed no significant difference between three groups of patients treated with lavage method alone, erythromycin injection alone, and their combination in terms of duration of endoscopy, the frequency of blood transfusions, the need for second endoscopy, the number of transfused blood units, and mortality rate on days 2, 7, and 30. Moreover, the erythromycin injection alone revealed the same effects (5). On the contrary; another study indicated the beneficial effect of gastric lavage on decreasing the endoscopy time (6). According to one study, the effect of lavage on the improvement of endoscopic vision depends on the location of lesion, and this effect is noticeable in the fundus lesions (7). Other studies have investigated the effect of solvent temperature used in lavage and found out no difference between 4°C and 37 °C saline solutions in terms of controlling dogs ‘gastric bleeding in vitro (8). 

Unfortunately, some studies have reported the nasogastric intubation-induced complication of gastric lavage in more than one-third of the patients (2). Some studies held much more pessimistic view regarding nasogastric intubation. A review study, for example, noted that gastric lavage or aspiration cannot be normally recommended based on available resources unless a randomized, well-designed, and large trial (which is not available now) reaches other findings. 

Nasogastric intubation has also been regarded as a painful and time-consuming solution with no beneficial results for patients. In this regard, the use of other clinical and laboratory parameters and less invasive measures such as pre-endoscopic erythromycin infusion is an appropriate and better alternative to improve the endoscopic gastric vision (9). 

 These cases require more investigations on gastric lavage or gastric lavage with other solutions. Additionally, saline may yield better results, particularly if a substance could reduce the need for emergency endoscopy and help control bleeding in areas where there is no quick access to endoscopy. Hemostasis powder^®^ is a new plant emulsion being developed through the joint cooperation of the departments of pharmacology and digestion and medical faculty in Iran. The drug was approved by Iran’s Food and Drug Administration and the Ministry of Health and Medical Education. The powder can be effective in controlling bleeding. An important feature of this drug is its acceptable strength, reasonable price, and ease of use. Previous studies have also documented such an effect in animal studies (10) as well as its beneficial effect on endoscopic control of bleeding from peptic ulcer in human studies (11). An interesting feature of this drug and similar drugs in terms of method (TC-325 hemostatic powder) is the need for no injections at the bleeding site to treat bleeding. This led us to develop a pilot study to examine the effect of gastric lavage with this powder to evaluate its effect on the primary control of gastrointestinal bleeding and the need for endoscopic therapeutic intervention.

## Methods

This clinical trial was carried out in the Emergency Department of Ghaem and Imam Reza Hospitals in Mashhad during 2017-2018. At the beginning of the study, the study methodology and objectives were explicitly described to the participants and their informed consent was then obtained. Patients who referred to the emergency department are those due to UGIB without coagulation disorder (with INR and normal platelet) and had no history of using anticoagulants such as warfarin and antiplatelet drugs. All patients who complained of hematemesis with Glasgow-Blatchford bleeding score (GBS) >1 were included in the study to have equal bleeding severity and had hemodynamic instability (resting heart rate >100, systolic pressure <90 mm hg or postural orthostatic tachycardia syndrome (POTS). In this study, resuscitation measures were first taken to stabilize the patients’ general conditions, and the gastric lavage was then carried out using a nasogastric tube and normal saline. According to the researcher's prediction of differences based on the “ lack of need for endoscopy treatment during primary endoscopy”  an Equivalent to 0.4 and   based on 20% loss and based on alpha 0.05 and a beta of 0.2 sample size was calculated for 30 individuals per group. But in this study (due to the need for research information to design future studies, after the entry of 54 patients (27 persons in each group), the analysis was performed.

First, routine emergency procedures were adopted for each patient in each group, and then gastric lavage continued in the control group until was a clear fluid discharge out of the stomach. 

***Intervention in the experimental group*** *: *In the experimental group, the gastric lavage was carried out using the hemostasis powder^® (^An herbal drug that was first registered with Samen-Ista brand and then renamed). Hemostasis powder^®^ is a new plant emulsion being developed through the joint cooperation of the Department of Pharmacology and Digestion and medical faculty at Mashhad University of Medical Sciences, Mashhad, Iran. 

The drug was approved by Iran Food and Drug Administration and the Ministry of Health and Medical Education. Each package of hemostatic powder contains two cans of Tabashir and Mazo powders. To carry out the gastric lavage, the patient's stomach was first washed with 250 ml normal saline. Then the first (white) powder was dissolved with 100 ml normal saline and slowly entered into the stomach through a nasogastric tube. About 15minutes later, the second (brown) powder was dissolved with normal saline (100 cc), slowly injected into the stomach similar to the previous solution, and then discharged out of the stomach15 minutes later.

***Primary outcome: ***All patients in both of the groups were treated with the same procedure stabilize vital signs (normal saline and blood infusion if needed). In the case of unstable vital signs remained following the above treatment, emergency endoscopy was performed. In this case, if the vital signs persisted, the endoscopy was performed 24 hours after referral. Finally, the patients were compared in terms of re-bleeding, time needed for endoscopy, quality of endoscopy, and need for intervention during endoscopy. 

The quality of endoscopy vision was assessed using a 5-point scale (with 1 indicating a low quality and 5 representing a high quality). The need for blood transfusion (Hb <7g / dl) was assumed for most patients to maintain blood level at 7g / dl. This threshold was considered as (Hb<9g / dl) for cardiac high-risk patients. Furthermore, there was a need for blood transfusion despite normal levels of hemoglobin in patients with active bleeding and hypovolemia and with no hemodynamic correction after saline injection (2 liters).

Rebleeding was defined as an apparent hematemesis; more than two units decrease in the hemoglobin level within 24 hours after primary endoscopic homeostasis, and shock (as a systolic blood pressure<90 mmHg or higher heart rate >110 beats / min) in the presence of continued melena. The patients' information was collected while observing ethical considerations, and the collected data were analyzed using SPSS software version 18. A p<0.05% was set as the significance level in all statistical tests. This study was extracted from a clinical trial approved by the Ethics Committee of Mashhad University of Medical Sciences with the code of ethics IR.mums.REC.1395.335, registered in Iranian Registry of Clinical Trials with IRCT No.20140824018915N5

## Results

The present study was carried out on 54 patients with UGIB. Hemostatic powder was used for gastric lavage in 27 patients. The gastrointestinal bleeding was observed in 63% and 70% of the subjects aged above 50 years in the control and the experimental groups, respectively. In this regard, there was no significant difference between the two groups in terms of their mean age (table 1).

**Table 1:** Initial patient information

**Pvalue**	**Intervention (n=27)**	**Control (n=27)**	**Number**
0.85	57.3±12.8	56.6±16.1	Age (year)
0.6	15(56%)	18(67%)	Male N(%)
0.78	Ulcer with oozing (FC I) : 12 pigmented hematin on ulcer base (FC II): 5 visible vessel (FC II): 4 adherent clot (FC II):5 esophageal varices : 1	Ulcer with oozing (FC I) : 9 pigmented hematin on ulcer base (FC II): 7 visible vessel (FC II): 5 adherent clot (FC II):4 esophageal varices : 2	Endoscopy lesion
0.69	Esophagus: 1 Duodenum: 6 Cardia :4 Fundus: 4 Body: 7 Antrum: 5	Esophagus: 2 Duodenum: 5 Cardia :7 Fundus: 3 Body: 6 Antrum: 4	Location of lesion

The results showed that the endoscopy lasted for 1.08±4.21 minutes in the experimental group and 9.29±3.04 minutes in the saline group; the difference was significant (*p*=0.001). There was also a need for endoscopy 8.04±4.83 and 6.73±14.12 hours after referral in the saline and hemostatic powder lavage groups, respectively (*p*=0.001). It is noteworthy that 59.2% and 18.5% of the patients undergoing gastric lavage with homeostasis powder and saline required no treatment during initial endoscopy, respectively (*p*=0.002). Furthermore, the hemostasis powder resulted in a significant improvement in the endoscopic vision and the quality of endoscopy (4.1 ver. 2.3). There was no difference between the two groups in terms of their need for blood transfusion. Re-bleeding occurred in two patients (not undergoing endoscopy treatment) in the powder group, and endoscopy treatment with hemostasis powder^®^ and adrenaline injection were successfully carried out at the ulcer site in the hemostatic powder group (table 2).

**Table 2:** Treatment outcomes after lavage

P value	Hemostatic powder lavage	Saline lavage	
0.001	4.21±1.08	9.29±3.04	Duration of endoscopy (min)
0.001	14.12±6.73	8.04±4.83	Time required for endoscopy (hours sine referral)
0.04	4.1	2.3	Quality of endoscopy
0.4	1.02±1.69	1.35±1.78	Need for blood transfusion (blood bag)
0.00002	16(59.2%)	5 (18.5%)	Lack of need for endoscopy during primary endoscopy
0.2	2 (7.4%)	1(3.7%)	Rebleeding
	0	0	30-day mortality

## Discussion

Gastric lavage has been one of the main pillars of the classic treatment for gastric bleeding for many years (12) and is commonly used in treating patients with gastrointestinal bleeding; however, its effectiveness has been questioned with regard to its painful intubation process. Few studies have examined the effectiveness of gastric lavage in treating gastrointestinal bleeding, and also a limited number of studies have addressed the role of gastric lavage using specific substances in the treatment of gastric bleeding. These studies were single-blind, randomized, and prospective studies and compared the effects of use and non-use of gastric lavage. This study researched the effect of lavage on the accuracy of physicians' speculations about the risk of lesion, rebleeding, and mortality rate, and the results suggested that it had significant effect on any of the aforementioned variables. Furthermore, 34% of the patients experienced pain, nasal bleeding, or failed nasogastric intubation; hence, the present study did not support the nasogastric intubation in the treatment of gastric bleeding (2). Another study investigated the effect of saline temperature on gastric lavage. In this study, dog stomachs were ulcerated mechanically in vitro. The bleeding rate was measured in the gastric lavage with normal saline at 37 °C and compared with gastric lavage with 4° saline with or without added norepinephrine. It was later concluded that low-temperature saline was not more effective than 37° C saline in reducing gastric bleeding. These data also did not support the effect of norepinephrine on gastric lavage to control stomach bleeding (8). Another study found out that the gastric lavage had no effect on mortality, length of stay, or blood transfusion; however, NGL was associated with a reduction in endoscopy duration and an increased risk of aspiration (OR 2.69, CI 95 %, 1.08-6.73) (6). Moreover, another study showed a direct relationship between the lesion site and the effect of gastric lavage on endoscopic quality. 

This study was carried out on 39 patients, and the results showed that lavage had no significant effect on the quality of endoscopy vision in esophageal, gastric antrum, or duodenum lesions; however, gastric lavage provided significantly the higher quality of endoscopy for fundus lesions *(P*=0.02). 

There was no significant difference between the groups in terms of determining the source of bleeding, achieve homeostasis, rebleeding, the need for re-endoscopy, and mortality Moreover, no specific complication was reported in this study. As an exception, a patient had experienced failed nasogastric intubation (7). Similarly, Dr. Tavakoli et al. investigated the effect of gastric lavage with tranexamic acid (TXA). 

In this randomized, double-blind clinical trial, which included 410 patients with acute gastrointestinal bleeding, all patients received conventional treatment. Subjects were divided into three groups: (A) 138 patients with TXA intravenous injection (1g qg6h); (B) 133 patients with topical TXA (1g single dose vi nasogastric tube) with systemic TXA; and (C) 139 patients with place before 24 hours (sodium chloride 0.9%). Subgroup statistical analysis was performed for emergency endoscopy, mortality, re-bleeding, blood transfusion, endoscopic and / or surgical interventions, and health status. These researchers stated that endoscopy duration was significantly reduced in Group C (p<0.001). There was no significant difference between the treatment groups in terms of mortality rate, re-bleeding, blood transfusion, and the incidence of endoscopic and / or surgical interventions (13). The present study addressed the effect of the hemostasis powder® on gastric lavage. This powder was initially licensed under the brand (Samen-Ista), and relevant animal studies were carried out accordingly. After mechanically cutting the rats’ tails and the rabbits’ ears and creating gastric bleeding ulcers in the phase of animal study, its effect on bleeding control was investigated, and its positive effect was then revealed (10). After obtaining the necessary permissions, relevant human studies were carried out. The first study examined its effect on controlling bleeding using other common therapies, and its positive effects were then published (11). 

Such effects led the researcher of the present study toward the idea that gastric lavage with this low cost drug can manage bleeding, reduce the need for emergency endoscopy, and improve the clinical outcome of gastric bleeding, especially in areas where endoscopy is unavailable. The results also indicated that gastric lavage with this powder might reduce the frequency of the need for endoscopy treatment during the early endoscopy in many cases as such 59.2% and 18.5% of patients in the hemostatic powder and saline groups required no treatment measure during the early endoscopy (P=0.002), suggesting that the gastric lavage with this powder can reduce the need for emergency endoscopy and convert an emergency condition into an elective one. There was also a need for endoscopy 8.04±4.83 and 6.73±14.12 hours after referral, one average, in hemostasis powder^® ^lavage and saline lavage group, respectively (P=0.001). Such an interval is of great importance in areas where endoscopy is not available. This drug is an invented drug manufactured in Iran, which is currently undergoing pilot studies; therefore, it cannot be directly compared to other studies. 

The only point in this regard is that it is similar to other drugs in terms of method of use (not production method and drug ingredients).They are called hemospray and hemostatic powder (TC-325) is the most common among them. Hemospray (TC-325, Cook Medical, United States) is an inert mineral-based compound, which, in contact with blood, absorbs water and acts cohesively and adhesively, thereby forming a covering mechanical tamponade. By fluid absorption, TC-325enhances clot formation by deforming and packing erythrocytes, concentrates activated platelets with clotting factors and interacts with the fibrin matrix (14); within 24 to 72 h, the adherent coat sloughs off into the GI lumen (15). With such local hemostatic properties, studies suggest that TC-325is equally effective in patients with and without use of systemic antithrombotic therapy (16).

In a review study, Faccirusso et al. investigated the therapeutic effect of Hemospray on gastrointestinal bleeding. In their study, 24 studies, of which three were randomized-controlled trials, with 1063 patients were included in the meta-analysis. Immediate hemostasis was achieved in 95.3% of patients, with no difference based on the adopted treatment strategy, hemostatic agent, and bleeding etiology. Success rate was slightly lower in spurting bleeding. Hemostatic powders had effectiveness similar to the conventional endoscopic therapy (P=0.9). 

Thirty-day rebleeding rate was 16.9% (9.8%-24%), not differing from other endoscopic treatments (P=0.55) (17). It should be noted that no study has dealt with gastric lavage drug (with regard to the administration method) yet.In conclusion gastric lavage with hemostasis powder^® ^is a useful measure in the early treatment of patients with upper gastrointestinal bleeding and no upper endoscopy to control bleeding in severe cases and can convert emergency endoscopy into diagnostic elective endoscopy with higher quality. Accordingly, it is recommended to be used in emergency rooms and pre-hospital health centers for primary health care in rural areas before the disease spreads. 
